# Garlic Improves Normal Water and Sodium Handling in Diabetic Kidneys by Attenuating Atrial Natriuretic Peptide: A Comparison with Metformin Treatment

**DOI:** 10.3390/ijms27177935

**Published:** 2026-09-06

**Authors:** Abdulwahab K. Aldousar, Amani M. Al-Adsani

**Affiliations:** Department of Biological Sciences, Faculty of Science, Kuwait University, P.O. Box 5965, Safat 13060, Kuwait

**Keywords:** diabetes mellitus, garlic, metformin, atrial natriuretic peptide, polyuria, rat

## Abstract

Diabetes mellitus (DM) is a chronic endocrine abnormality characterised by impaired insulin function and systemic hyperglycaemia, which alter fluid balance and hormonal regulation. The cardiac hormone atrial natriuretic peptide (ANP) modulates renal natriuresis and diuresis, and its expression is disrupted in DM. We evaluated the effects of oral aqueous garlic extract (GE) on physiological, biochemical, and molecular parameters in streptozotocin-induced type 1 diabetic male Sprague–Dawley rats, using metformin as a positive control. Parameters assessed included fasting blood glucose (FBG), body weight (BW), fluid intake, urine output, renal sodium excretion, serum ANP concentration, and right atrial ANP and renal NPR-A gene expression via qRT-PCR. GE significantly reduced FBG by 64.8%, attenuated polyuria and polydipsia, and improved BW. GE also downregulated right atrial ANP gene expression by 56.2% and reduced serum ANP concentrations by 34.6%, which occurred alongside a significant reduction in urinary sodium excretion. These findings suggest that GE-induced improvements in fluid and electrolyte balance in type 1 DM are partly mediated through modulation of the ANP signalling pathway.

## 1. Introduction

**Figure 1:**
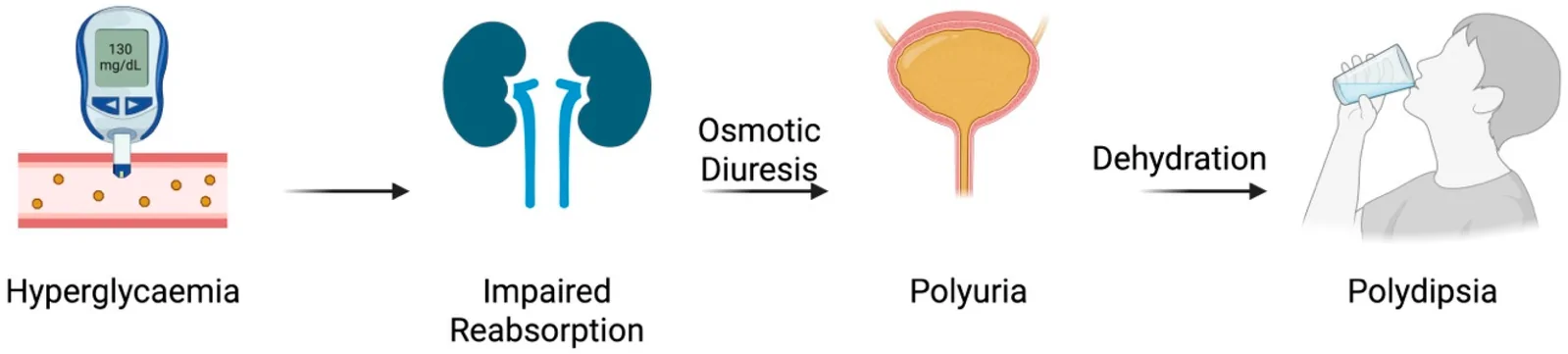
Development of polyuria and polydipsia in DM. Elevated circulating glucose exceeds the resorptive capacity of glucose transporters in the proximal tubule, causing excess glucose to pass into the distal nephron. The presence of glucose in the tubular lumen disrupts the normal osmotic gradient, preventing water reabsorption into the medullary interstitium and retaining it within the collecting duct. This osmotic diuresis induces systemic dehydration, subsequently triggering compensatory polydipsia. Created with BioRender.com. Retrieved from https://BioRender.com/c1ntdtb on 4 July 2026.

Diabetes mellitus (DM) is a chronic metabolic disorder primarily characterised by persistent hyperglycaemia resulting from impaired glucose homeostasis [1]. This hyperglycaemia is caused either by insufficient insulin production and release (type 1 DM) or by cellular insulin resistance (type 2 DM). Specifically, type 1 DM predominantly results from the autoimmune destruction of pancreatic beta cells (β-cells), leading to decreased insulin production. DM is associated with severe physiological, biochemical, and metabolic complications. These complications include abnormal hormonal regulation and subsequent fluid and electrolyte imbalances driven by osmotic diuresis [2,3]. Osmotic diuresis occurs when excess glucose filtration exceeds the tubular maximum for glucose, thereby reducing water reabsorption in the tubules and collecting ducts; this ultimately results in polyuria, natriuresis, and compensatory polydipsia [4,5,6]. This altered fluid state activates multiple hormonal systems responsible for preserving fluid and electrolyte homeostasis, such as the antidiuretic hormone (ADH) system and the renin–angiotensin–aldosterone system (RAAS), both of which are upregulated during diabetic dehydration [7,8,9]. Figure 1 illustrates the development of these symptoms in response to hyperglycaemia.

**Figure 2:**
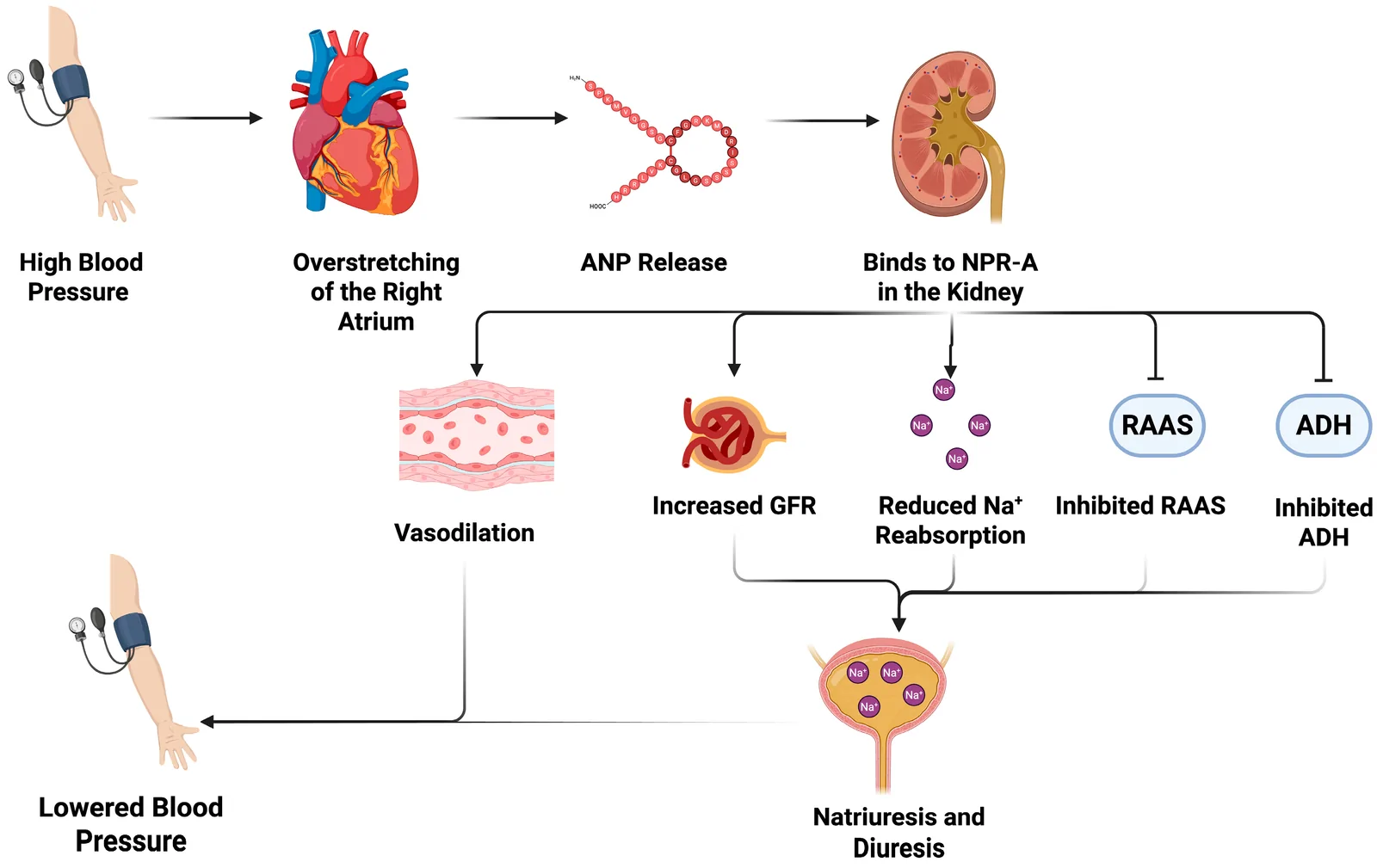
Haemodynamic regulation by ANP. Elevated blood pressure and volume lead to the mechanical stretching of the right atrium. This stretching stimulates the release of ANP into the circulation. Upon binding to the renal NPR-A receptor, ANP increases the Glomerular Filtration Rate (GFR), attenuates sodium reabsorption, and inhibits the RAAS and ADH pathways, culminating in natriuresis and diuresis. Concurrently, ANP induces vasodilation; collectively, these mechanisms reduce systemic blood pressure. Created with BioRender.com. Retrieved from https://BioRender.com/b6g549r on 4 July 2026.

Conversely, the atrial natriuretic peptide (ANP) system plays a vital counter-regulatory role in fluid and electrolyte homeostasis, opposing the effects of ADH and RAAS. ANP is a cardiac hormone primarily secreted by myocytes in the posterior wall of the right atrium in response to mechanical stretching caused by increased venous return [10,11]. Upon release, ANP promotes natriuresis, diuresis, and vasodilation, thereby suppressing the opposing effects mediated by the RAAS and ADH. These downstream actions are mediated by ANP binding to its homologous NPR-A receptor, which catalyses the conversion of guanosine triphosphate to cyclic GMP. This activation initiates a signalling cascade that increases the glomerular filtration rate and drastically attenuates sodium reabsorption [12,13,14,15]. Figure 2 illustrates the physiological actions of ANP.

The alterations in the cardiac synthesis, release, and renal action of ANP during DM are complex and stage-dependent; consequently, findings from previous studies remain inconsistent. These fluctuations are likely related to disease progression; for example, during the early stages of DM, reduced plasma ANP levels have been reported and may serve as a predictive marker for disease onset [16]. This early-stage reduction in ANP may be caused by impaired cardiac or renal signalling pathways, and by increased oxidative stress [17,18,19]. Conversely, as DM progresses, elevated plasma and tissue ANP levels are observed. This elevation likely results from fluid retention and increased atrial mechanical stretching or acts as a compensatory mechanism against the heightened activity of the RAAS and ADH [20,21,22]. These stage-dependent variations in ANP levels highlight the dynamic regulatory role of this hormone throughout the progression of DM, suggesting its potential as a therapeutic target.

Natural herbs, compounds, and extracts are attracting increasing interest as complementary or alternative therapies for treating or mitigating various medical conditions, including DM. One such herb is garlic (*Allium sativum* L.), which has been used in traditional medicine for millennia across various regions. Specifically, regarding DM, garlic has shown promise in managing hyperglycaemia and its systemic complications. Its diverse bioactive sulphur-containing compounds—such as allicin, S-allyl cysteine, and diallyl disulphide—exhibit insulinotropic, antioxidant, and anti-inflammatory effects [23,24,25,26]. Experimental studies in STZ- and alloxan-induced diabetic rats have demonstrated that garlic extract increases insulin secretion and circulating levels, thereby reducing blood glucose and mitigating the systemic complications of DM, such as polyuria and polydipsia [27,28,29].

Despite extensive research investigating the effects of garlic in DM, little is known about its impact on ANP expression and signalling, and its subsequent effect on the renal handling of sodium and water and overall fluid dynamics. Given the central role of ANP in fluid and sodium homeostasis and its fluctuating levels in DM, determining whether garlic extract modulates ANP pathways could elucidate its mechanisms in attenuating diabetic natriuresis and polyuria. To our knowledge, no prior studies have directly assessed the influence of garlic on atrial ANP gene expression, circulating ANP levels, and renal ANP receptor distribution and density in diabetic conditions.

Therefore, this study aimed to investigate the effects of an aqueous garlic extract (GE) on right atrial ANP gene expression, serum ANP concentration, and renal ANP receptor density in streptozotocin (STZ)-induced diabetic rats as an experimental model of type 1 DM. In addition to molecular markers, we evaluated physiological indicators of glycaemic control and fluid balance. These included fasting blood glucose (FBG), body weight (BW), food intake (FI), water intake (WI), urine output, serum sodium concentration (SSC), and urine sodium output (USO). Ultimately, this study aimed to explore whether garlic restores the normal renal handling of sodium and water by modulating ANP expression, release, and receptor density in DM.

## 2. Results

### 2.1. Effect of Oral GE Treatment on Physiological Parameters

**Figure 3:**
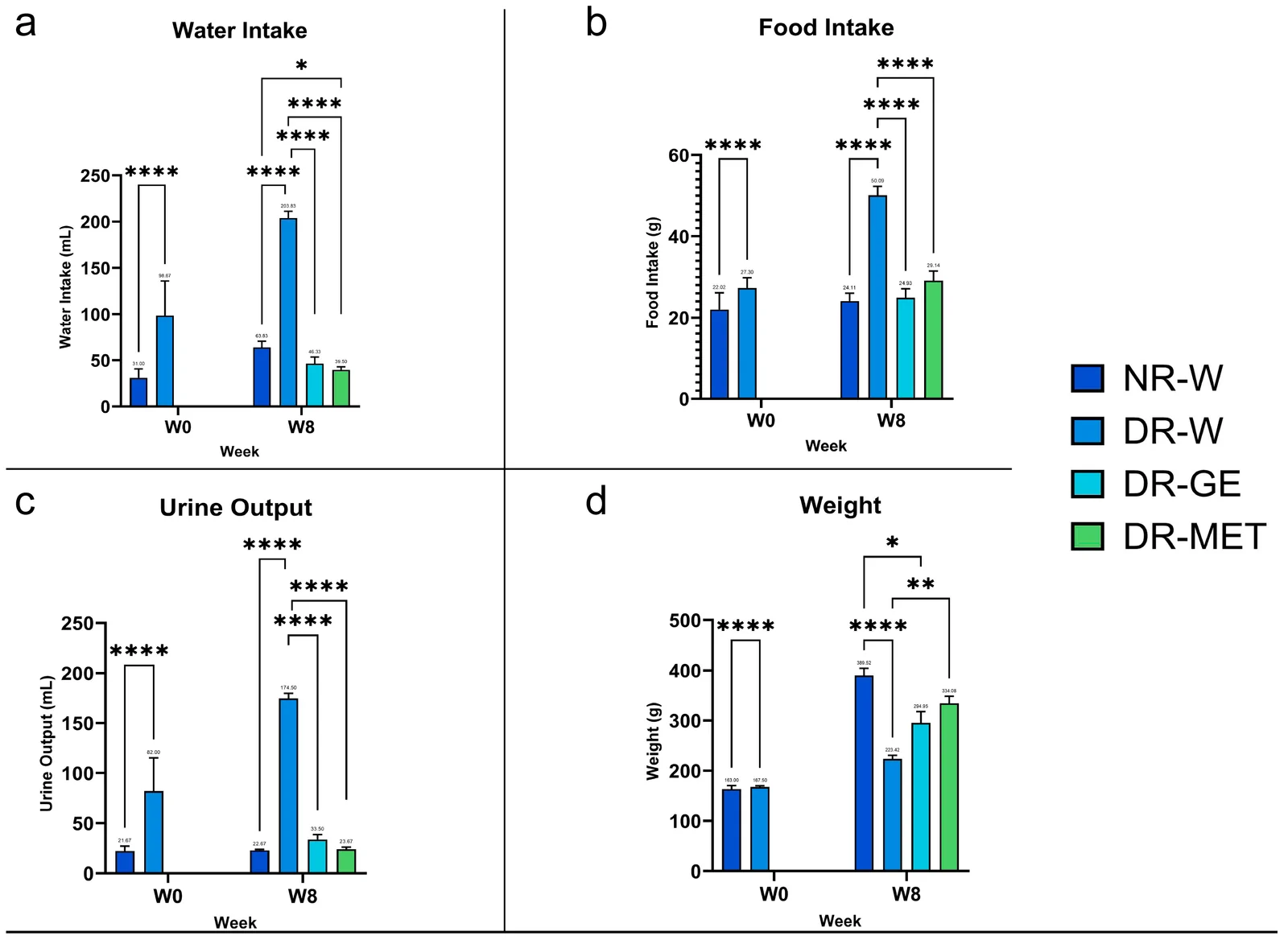
Effect of oral GE treatment on the physiological parameters of diabetic rats at W0 and W8. (**a**): Water intake (mL/day). (**b**): Food intake (g/day). (**c**): Urine output (mL/day). (**d**): Weight (g). Data are expressed as the mean ± SEM. * *p* ˂ 0.05, ** *p* ˂ 0.01, **** *p* < 0.0001 (*n* = 3 at W0; n = 6 at W8).

Compared with the NR-W group, the DR-W group exhibited significantly increased WI, FI, and UO at W0, and these increases persisted until W8. Following eight weeks of GE treatment, the DR-GE group demonstrated significant decreases (*p* ≤ 0.0001) in WI (77.3%), FI (46.9%), and UO (80.8%) compared with the DR-W group. Specifically, the mean values for WI, FI, and UO in the DR-GE group were 46.33 mL, 24.93 g, and 33.50 mL, respectively, whereas the DR-W group averaged 203.83 mL, 50.09 g, and 174.50 mL, respectively. The effects of GE on these parameters were comparable to those observed in the DR-MET positive-control group, which exhibited mean values of 39.50 mL, 26.62 g, and 23.67 mL, respectively (Figure 3a–c).

Regarding BW, no significant difference was observed between the DR-W and NR-W groups at W0; however, the DR-W group exhibited a significant decrease in BW by W8. GE treatment significantly increased the BW of the DR-GE group by 32% (*p* ≤ 0.0001), reaching a mean of 294.95 g compared with 223.42 g in the DR-W group. This recovery was comparable with the BW observed in the DR-MET positive-control group (mean: 334.08 g) (Figure 3d).

### 2.2. Effect of Oral GE Treatment on Fasting Blood Glucose

**Figure 4:**
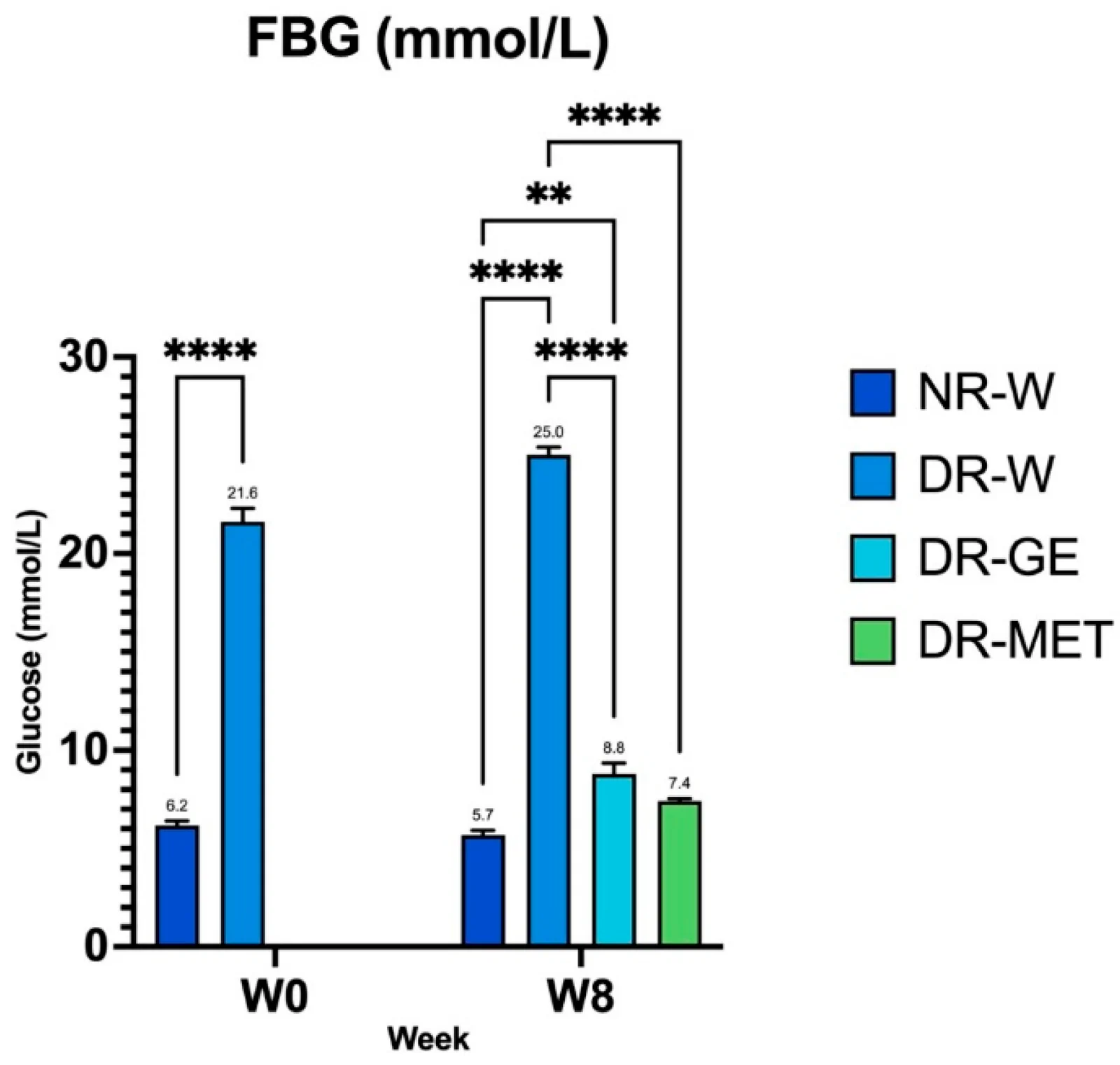
Effect of GE treatment on fasting blood glucose (FBG) levels after eight weeks. Oral GE treatment significantly reduced FBG levels in diabetic rats, yielding an effect comparable with that of the metformin positive control. Data are expressed as the mean ± SEM (*n* = 6 per group). ** *p* < 0.01, **** *p* ˂ 0.0001.

The FBG levels in the DR-W group were significantly higher than those in the NR-W group at W0 and W8. Following eight weeks of GE treatment, FBG levels in the DR-GE group decreased significantly by 64.8% (mean: 8.8 mmol/L) compared with the DR-W group (mean: 25.0 mmol/L) (*p* ≤ 0.0001). Furthermore, the FBG levels in the DR-GE group did not differ significantly from those in the DR-MET group (mean: 7.4 mmol/L) (Figure 4).

### 2.3. Effect of Oral GE Treatment on Right Atrial ANP and Renal NPR-A Gene Expression

**Figure 5:**
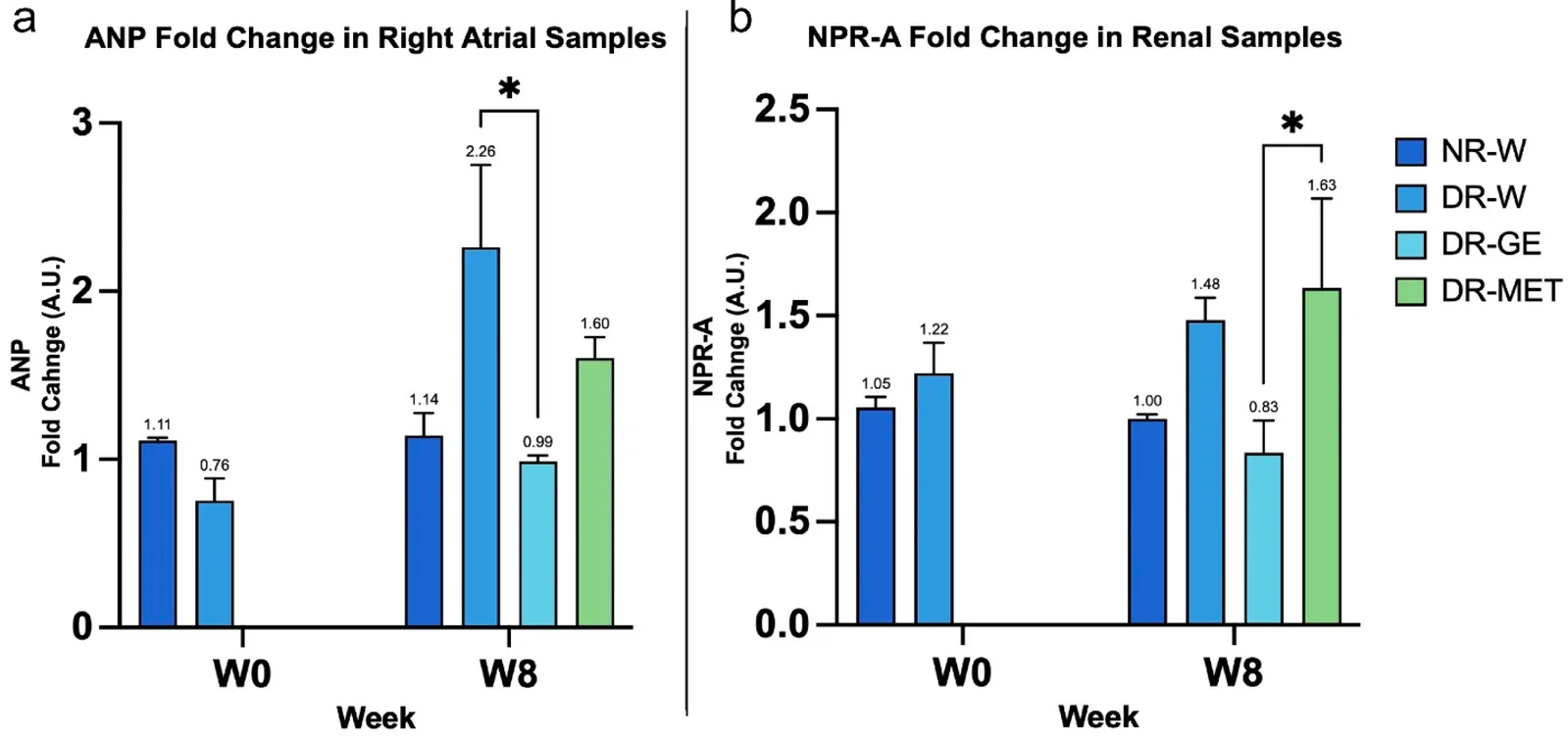
Relative fold changes in gene expression following treatment with GE or metformin. (**a**): Right atrial ANP gene expression. (**b**): Renal NPR-A gene expression. The figure bars represent mean ± SEM With a significant level of * *p* ˂ 0.05. *n* = 3.

At W0, right atrial ANP gene expression did not differ significantly between the DR-W and NR-W groups. By W8, right atrial ANP expression in the DR-W group exhibited a substantial upward trend compared with the NR-W group, though this difference did not reach statistical significance. GE treatment significantly decreased ANP expression in the DR-GE group by 56.2% (*p* ≤ 0.05), reducing the mean fold change to 0.99 compared with 2.26 in the DR-W group. In contrast, metformin elicited a weaker, statistically non-significant reduction in ANP expression in the DR-MET group (mean fold change: 1.6) (Figure 5a).

Similarly, renal NPR-A gene expression did not differ significantly between the DR-W and NR-W groups at W0. By W8, renal NPR-A expression demonstrated a non-significant upward trend in response to the induction of diabetes (DR-W group) and a subsequent non-significant downward trend following GE treatment (DR-GE group). Interestingly, metformin treatment significantly elevated NPR-A expression compared with GE treatment (Figure 5b).

### 2.4. Effect of Oral GE Treatment on Serum ANP Concentration

**Figure 6:**
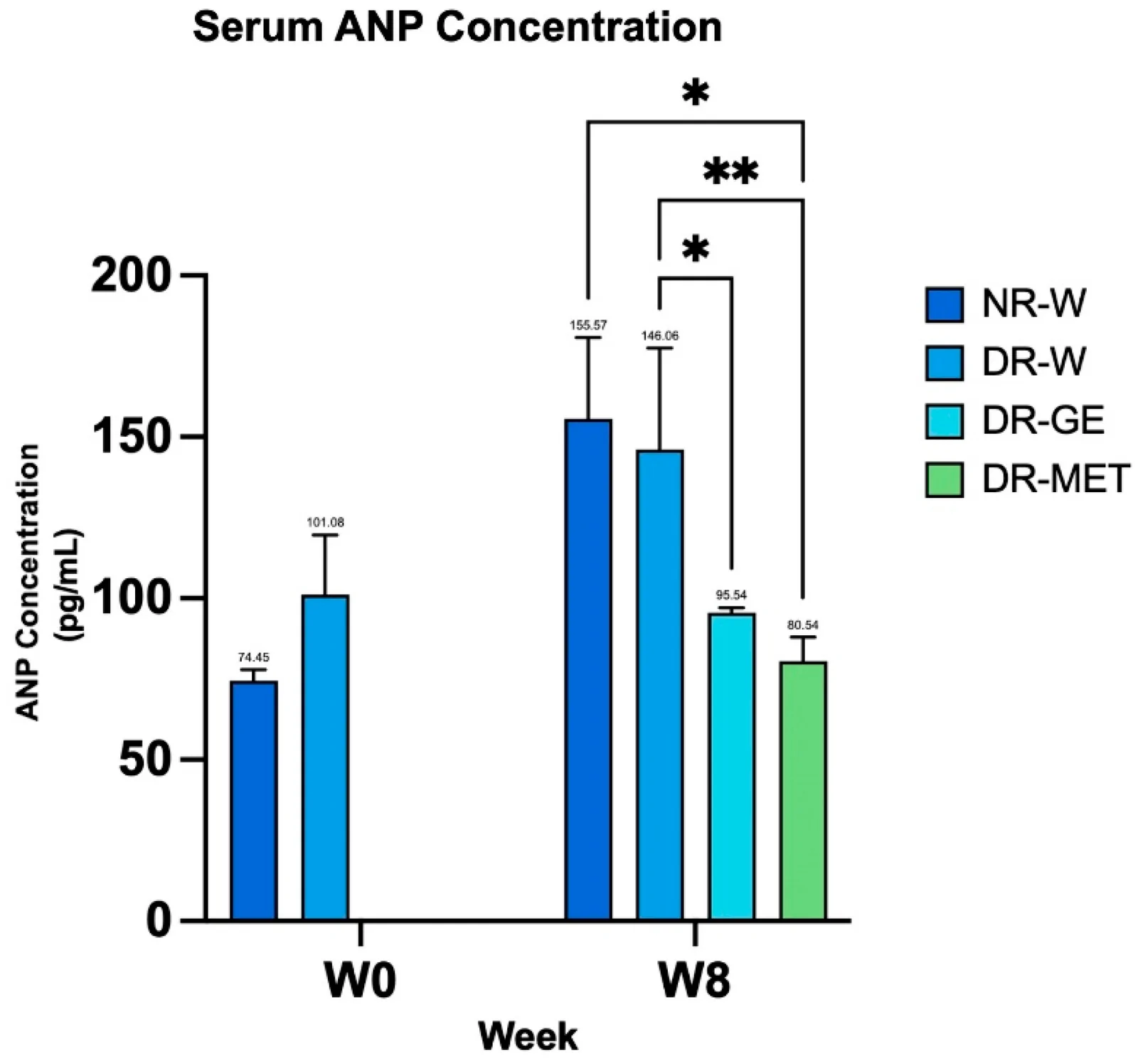
Effect of oral GE treatment on serum ANP concentration. Data are expressed as the mean ± SEM (*n* = 3 per group). * *p* ˂ 0.05, ** *p* ˂ 0.01.

Serum ANP concentration did not differ significantly between the DR-W and NR-W groups at W0 or W8. In contrast, GE treatment significantly decreased serum ANP concentrations in the DR-GE group by 34.6% (mean: 95.54 pg/mL) compared with the DR-W group (mean: 146.06 pg/mL) (*p* ≤ 0.05). This reduction was comparable with that induced by metformin; serum ANP levels in the DR-MET positive-control group averaged 80.54 pg/mL (Figure 6).

### 2.5. Effect of Oral GE Treatment on Serum Sodium Concentration and Urine Sodium Output

**Figure 7:**
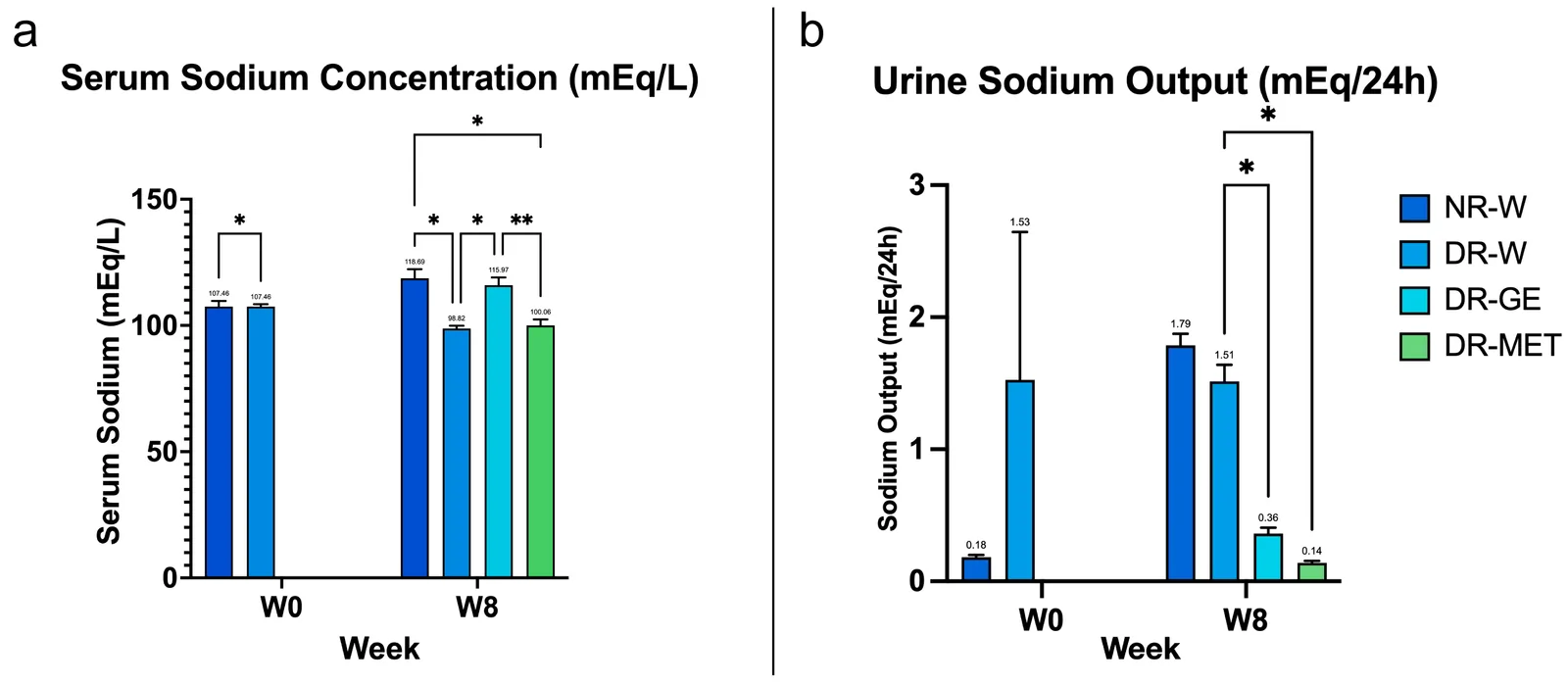
Effect of GE and metformin on (**a**): SSC and (**b**): USO. Sodium concentrations were quantified via ICP-MS. At W8, SSC fluctuated among the groups without a definitive directional pattern. Conversely, USO was markedly reduced following GE or metformin treatment. Data are expressed as the mean ± SEM (*n* = 3 per group). * *p* ˂ 0.05, ** *p* ˂ 0.01.

At W0, ICP-MS analysis revealed comparable SSC values between the NR-W and DR-W groups, indicating effective homeostatic regulation. Although statistically significant differences in SSC were observed among the respective groups at W8, these fluctuations lacked a consistent directional trend (Figure 7a).

Regarding USO, its levels showed a non-significant upward trend in the DR-W group in comparison to the NR-W group at W0; however, this difference resolved by W8. GE treatment significantly reduced USO in the DR-GE group (*p* ≤ 0.05) by 76.2% (mean: 0.36 mEq/24 h) compared with the DR-W group (mean: 1.51 mEq/24 h). Furthermore, USO expression in the DR-GE group did not differ significantly from that in the DR-MET positive-control group (mean: 0.14 mEq/24 h) (Figure 7b).

## 3. Discussion

This study investigated the role of oral GE treatment in modulating the ANP system—a key hormonal regulator of water and sodium homeostasis—in type 1 DM. Specifically, we focused on changes in key physiological and biochemical complications associated with DM, including polyphagia, polydipsia, polyuria, BW loss, hyperglycaemia, and altered insulin levels, alongside SSC and USC. Furthermore, we assessed molecular alterations in ANP and ANP receptor messenger RNA (mRNA) expression in the right atrium and kidneys following GE treatment.

The STZ-induced diabetic rat model utilised in this study successfully developed the characteristic symptoms mentioned above, consistent with the classic presentation of type 1 DM reported previously [23,24,27]. These symptoms arise as complications of insulin deficiency, which reduces peripheral glucose uptake and causes subsequent glucosuria-driven diuresis [7]. These systemic disruptions extend beyond glucose metabolism to alter overall fluid and electrolyte balance.

GE treatment substantially attenuated several of these diabetic parameters. Although FBG levels significantly declined in the DR-GE group, they remained higher than those in the NR-W group. These outcomes corroborate the established insulinogenic capability of garlic, which is mediated by the protective effects of its active sulphur-containing compounds on pancreatic β-cells [23,24,26] by β-cell neogenesis [30]. Furthermore, the antioxidant properties of garlic may alleviate oxidative stress-induced damage to insulin-producing cells, potentially contributing to the partial restoration of insulin levels [28,29].

Alongside glycaemic improvements, our findings demonstrate that GE significantly attenuates the diabetic symptoms of polydipsia and polyuria. The reductions in WI and UO are consistent with improved glucose management driven by elevated insulin levels. Furthermore, these reductions reflect enhanced fluid balance resulting from a decreased osmotic load in the renal tubules relative to the medullary interstitium. These findings support the hypothesis that the systemic effects of garlic extend beyond insulin signalling to involve broader hormonal networks, such as the RAAS and ANP systems [27,31,32]. Regarding the RAAS, we have previously shown that garlic effectively ameliorates several components of this hormonal system [31,33].

The primary target hormone of this study was ANP, which is released by right atrial myocytes, plays a crucial role in sodium and water balance, and binds to its primary receptor, NPR-A [10,11,15]. We observed a modest, non-significant increase in right atrial ANP gene expression in the DR-W group, which aligned with the elevated, albeit non-significant, serum ANP concentrations observed in these untreated rats. This slight upregulation in expression may represent an early compensatory attempt to counteract volume overload; however, the lack of a corresponding significant increase in circulating ANP protein suggests that alternative compensatory mechanisms dominate at this disease stage. Interestingly, GE treatment significantly decreased both ANP mRNA expression and circulating peptide levels, reducing them to values numerically comparable to those observed in healthy controls. These novel findings highlight the efficacy of GE in ameliorating ANP dysregulation. Furthermore, this inverse relationship between insulin restoration and ANP modulation aligns with previous observations in diabetic models not treated with GE [34]. This interpretation is supported by previous research demonstrating that ANP levels decline in well-controlled DM or following the restoration of fluid balance [16,22]. Therefore, the GE-induced reduction in ANP likely reflects improved systemic homeostasis rather than a direct inhibitory effect on the ANP gene. Regarding the specific regulatory pathways, it is highly probable that GE, similar to metformin, reduces UO by attenuating natriuresis. This reduction in natriuresis, coupled with the primary insulin-mediated decrease in glucosuria, subsequently attenuates renal tubular osmotic pressure.

Although not statistically significant, a similar pattern was observed in renal NPR-A expression; diabetic rats exhibited an upregulation of NPR-A mRNA, which was subsequently reduced by GE treatment. This molecular behaviour of NPR-A and its response to GE mirrored the alterations observed in ANP levels. Conversely, metformin treatment significantly increased NPR-A expression. This observation indicates that GE and metformin may act via distinct receptor-level mechanisms, a hypothesis that could be substantiated by quantifying NPR-A protein expression.

The impact of GE treatment on serum sodium handling fluctuated among the groups without reflecting a definitive pattern. This likely reflects the innate capacity of the body to maintain electrolyte balance through multiple interacting compensatory mechanisms. Conversely, urinary sodium levels exhibited a trend aligning with ANP concentrations: the healthy and untreated diabetic groups displayed similarly high values, whereas the treated groups exhibited significantly lower values. Although consistent with ANP modulation, these findings raise questions regarding the physiological similarities between healthy and untreated diabetic rats in this specific context. Nevertheless, these results support the proposed inverse relationship between insulin and ANP, wherein increased insulin suppresses ANP and consequently reduces sodium excretion to restore homeostasis. These findings align with previous studies demonstrating that insulin directly suppresses ANP-driven natriuresis [35].

The mechanism by which GE affects ANP signalling is likely indirect and sequential. Enhanced insulin secretion and glycaemic control reduce the osmotic load and plasma volume disturbances that stimulate ANP release. Attenuating the stimulus for ANP release consequently downregulates the peptide, resulting in reduced sodium excretion and decreased diuresis. Although speculative, GE may also directly modulate the ANP system independently of insulin or via interactions with the RAAS and ADH pathways. Further studies are necessary to delineate these potential mechanisms. Furthermore, GE may interact with the RAAS [31,33] and ADH systems, both of which exert counter-regulatory effects on the ANP pathway [36,37].

**Figure 8:**
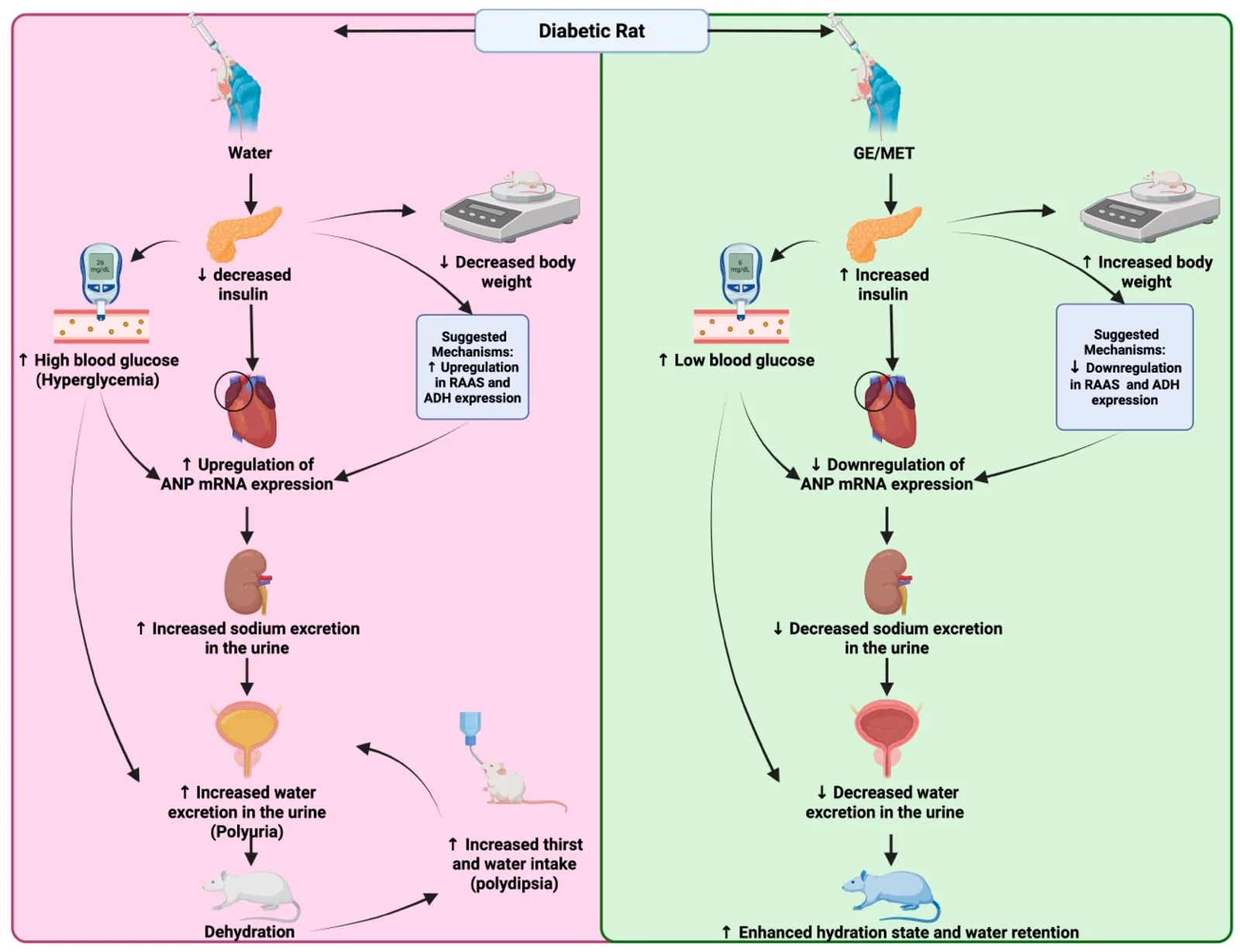
Proposed mechanism by which GE or metformin treatment modulates the ANP pathway to induce antidiuresis. In the untreated state, insulin deficiency upregulates ANP activity, culminating in polyuria and polydipsia. Treatment with GE or metformin increases insulin levels, which subsequently downregulates ANP activity and restores systemic hydration. Created with BioRender.com. Retrieved from https://BioRender.com/fzvlxf4 on 4 July 2026.

Figure 8 illustrates this proposed perspective, capturing the dynamics among metabolic control, hormonal regulation, and fluid balance. In the diabetic state, insulin deficiency leads to RAAS and ADH activation, increased ANP signalling, and elevated sodium and water loss. Conversely, GE or metformin treatment increases insulin levels or sensitivity, suppresses RAAS and ADH activity, and reduces ANP and NPR-A expression, ultimately restoring systemic fluid stability and attenuating polyuria. Future studies are required to further elucidate the effects of GE on other hormonal systems involved in fluid balance and metabolism.

## 4. Materials and Methods

### 4.1. Animals and Housing Conditions

Healthy male Sprague–Dawley rats (*n* = 36; initial BW, 160–180 g), descended from a colony originally procured from B & K Universal Co. (Hull, UK), were housed in the Animal Care and Breeding Facility at the Department of Biological Sciences, Kuwait University. The rats were kept in standard polypropylene cages (65 × 35 cm) with stainless-steel wire-mesh covers and lined with soft corn-sheaf bedding. During metabolic measurements, animals were housed individually in metabolic cages equipped with collection bottles at the base to capture all urine produced during the 24-h collection period. The animals were maintained under controlled environmental conditions (22–24 °C, 30–35% humidity, and a natural light/dark cycle). All rats had ad libitum access to a standard rodent diet (Mucedola, Settimo Milanese, Italy) and tap water. All housing conditions, study protocols, euthanasia methods, and sample collection and analysis procedures were performed in accordance with the Guide for the Care and Use of Laboratory Animals [38]. The experimental protocol was approved by the Institutional Research Ethics Committee at the Faculty of Science, Kuwait University (Approval No. SCI-IRB-2024/5-0490).

### 4.2. Experimental Animal Model

This study utilised both healthy rats and a rat model of STZ-induced type 1 DM. To induce type 1 DM, the experimental rats were fasted for 2 h and subsequently administered a single intraperitoneal injection of STZ (60 mg/kg BW; Sigma-Aldrich, St. Louis, MO, USA; Cat. No. S0130-1G) dissolved in 0.01 M sodium citrate buffer (pH 4.5). A separate group of control rats received an equivalent volume of the citrate buffer vehicle.

### 4.3. Measurement of Fasting Blood Glucose

Six days following the administration of STZ or the vehicle, initial FBG was measured in tail-vein blood samples using an Accu-Chek Performa glucometer (Roche Diagnostics GmbH, Mannheim, Germany). Rats with an FBG ≥ 16 mmol/L were classified as type 1 diabetic and included in the diabetic cohort, whereas those with an FBG ≤ 8 mmol/L were classified as healthy and assigned to the control group.

### 4.4. Preparation of the Aqueous Garlic Extract and Metformin Solution

Garlic (*Allium sativum* L.) bulbs, purchased locally, were peeled on crushed ice, homogenised in sterile 0.9% NaCl (150 g in 50 mL), and filtered. The homogenate was centrifuged at 15,000 rpm for 15 min at 4 °C. The stock extract was adjusted to a concentration of 500 mg/mL and stored at −80 °C. Daily aliquots were diluted 1:3 with distilled water prior to use. Metformin tablets (Glucophage^®^, 1000 mg; Merck KGaA, Darmstadt, Germany)) were ground and dissolved in distilled water to prepare a working concentration of 80 mg/mL. The active metformin content was verified according to the manufacturer’s specifications.

### 4.5. Experimental Groups and Oral Treatments

A total of 36 rats were randomly divided into six groups (*n* = 6 per group). The groups were designated as follows: healthy rats euthanised at week 0 (NR-W W0) and week 8 (NR-W W8) following water administration; diabetic rats euthanised at week 0 (DR-W W0) and week 8 (DR-W W8) following water administration; diabetic rats treated with GE and euthanised at week 8 (DR-GE W8); and diabetic rats treated with the metformin positive control and euthanised at week eight (DR-MET W8). Treatments were administered via oral gavage as a 0.5 mL bolus dose once daily at mid-morning, beginning after group allocation and continuing for eight weeks. GE was administered at a dose of 500 mg/kg BW, and the metformin solution was administered at 200 mg/kg BW daily. The water-treated control groups received an equivalent volume of water (0.5 mL/kg BW).

### 4.6. Assessment of Physiological and Biochemical Parameters

Initial and terminal FBG levels were measured at the designated time points. Furthermore, BW, FI, WI, and UO were recorded over a 24-h period every two weeks using metabolic cages. Concurrently, urine samples were collected to measure the urine sodium concentration (USC) via Quadrupole-Based inductively coupled plasma mass spectrometry (ICP-MS) (Model 7500a, RICCA, Arlington, TX, USA). The instrument was calibrated using a sodium ICP-MS standard of 1000 ppm diluted in 3% HNO3 (RICCA, USA). Readings were recorded in ppm using Qtegra ISDS software (version 2.10 SR2, Thermo Fisher Scientific, Waltham, MA, USA). At the end of the 8-week treatment period, the animals were anaesthetised using a 9:1 mixture of ketamine (10%; Dutch Farm Nedar, Host den Berg, Holland) and xylazine (10%; Interchemie, Venray, Holland) at a dose of 0.2 mL/100 g BW intraperitoneally, and blood was collected via cardiac puncture. Serum was separated and stored at −80 °C until the biochemical quantification of ANP using a commercial enzyme-linked immunosorbent assay kit (SPI Bio, Bertin Pharma, Montigny-le-Bretonneux, France).

### 4.7. Gene Expression Analysis

Total RNA from the right atrial tissue and the kidneys was extracted using the TriPure Isolation Reagent (Sigma-Aldrich, Zwijndrecht, The Netherlands) according to standard protocols. Kidney tissue was obtained as a cross-sectional slice (~50–100 mg) taken consistently across all animals and groups, ensuring proportional representation of cortical and medullary regions. RNA concentration and purity were assessed using a NanoDrop 8000 spectrophotometer (Thermo Fisher Scientific, Waltham, MA, USA). RNA integrity was evaluated using the Agilent RNA 6000 Nano Kit and an Agilent 2100 Bioanalyzer (Agilent Technologies, Santa Clara, CA, USA). Complementary DNA was synthesised using the SuperScript IV First-Strand Synthesis System (Life Technologies, Vilnius, Lithuania) according to the manufacturer’s protocol. Quantitative real-time PCR was performed using TaqMan gene expression assays on a ViiA 7 Real-Time PCR System, and data were analysed using QuantStudio Real-Time PCR Software (version 1.7.1, Applied Biosystems, Foster City, CA, USA). Equipment was housed at the Biotechnology Center (BTC), Faculty of Science, Kuwait University (Project GS01/02).

### 4.8. Statistical Analysis

A two-way analysis of variance was performed using GraphPad Prism version 9.2.0 (GraphPad Software, San Diego, CA, USA) to compare parameters including FBG, SI, FI, WI, UO, USO, SSC, and BW (n = 6 per group), as well as serum ANP levels and gene expression (n = 3 per group). Post hoc comparisons were conducted using the uncorrected Fisher’s least significant difference test. Results are expressed as the mean ± SEM. Differences between compared values were considered statistically significant at *p <* 0.05.

## 5. Conclusions

This study provides novel insights into the physiological effects of GE in an STZ-induced diabetic rat model, with a particular focus on ANP-mediated fluid regulation. The results reaffirm that GE treatment significantly attenuates hyperglycaemia and classic diabetic symptoms, including polyuria, polydipsia, and BW loss. Importantly, GE also reduced right atrial ANP gene expression, serum ANP concentrations, and urinary sodium excretion. These findings suggest that GE not only improves glycaemic control but also modulates hormonal and renal pathways critical to fluid and electrolyte homeostasis.

Compared with metformin, GE produced comparable improvements in several fluid regulation parameters, highlighting its potential as a complementary or alternative therapeutic agent. The modulation of ANP signalling may represent a key mechanism by which GE restores fluid balance in diabetic conditions.

Overall, this work supports the broader therapeutic profile of GE and encourages further investigation into its hormonal and renal actions in diabetes. Future studies should incorporate larger sample sizes, direct hormone pathway assessments, and the exploration of molecular mediators linking insulin sensitivity to natriuretic peptide regulation.

## Data Availability

Data described in the manuscript will be made available upon request pending application and approval. Requests should be directed to the corresponding author.

## Abbreviations

ADH	Antidiuretic Hormone
ANP	Atrial Natriuretic Peptide
BTC	Biotechnology Center
BW	Body Weight
DM	Diabetes Mellitus
DR-GE	Diabetic Rats treated with Garlic Extract
DR-MET	Diabetic Rats treated with Metformin
DR-W	Diabetic Rats treated with Water
FBG	Fasting Blood Glucose
FI	Food Intake
GE	Garlic Aqueous Extract
GFR	Glomerular Filtration Rate
ICP-MS	Inductively Coupled Plasma Mass Spectrometry
mRNA	Messenger RNA
NPR-A	Natriuretic Peptide Receptor-A
NR-W	Normal Rats treated with Water (control)
RAAS	Renin–Angiotensin–Aldosterone System
RNA	Ribonucleic Acid
SEM	Standard Error of the Mean
SSC	Serum Sodium Concentration
STZ	Streptozotocin
Tm	Transport Maximum
UO	Urine Output
USC	Urine Sodium Concentration
USO	Urine Sodium Output
W0	Week 0
W8	Week 8
WI	Water Intake
β-cells	Beta-cells
